# A Novel Assay Method to Determine the β-Elimination of Se-Methylselenocysteine to Monomethylselenol by Kynurenine Aminotransferase 1

**DOI:** 10.3390/antiox9020139

**Published:** 2020-02-05

**Authors:** Arun Kumar Selvam, Mikael Björnstedt

**Affiliations:** Department of Laboratory Medicine, Division of Pathology F46, Karolinska Institutet, Karolinska University Hospital Huddinge, 141 86 Stockholm, Sweden; arun.selvam@ki.se

**Keywords:** kynurenine aminotransferase 1, Se-methylselenocysteine, β-elimination activity, methylselenol, thioredoxin reductase, KYAT1 inducers, and KYAT1 inhibitors

## Abstract

Kynurenine aminotransferase 1 (KYAT1 or CCBL1) plays a major role in Se-methylselenocysteine (MSC) metabolism. It is a bi-functional enzyme that catalyzes transamination and beta-elimination activity with a single substrate. KYAT1 produces methylselenol (CH_3_SeH) via β-elimination activities with MSC as a substrate. This methylated selenium compound is a major cytotoxic selenium metabolite, causing apoptosis in a wide variety of cancer cells. Methylselenol is volatile and possesses extraordinary nucleophilic properties. We herein describe a simple spectrophotometric assay by combining KYAT1 and thioredoxin reductase (TrxR) to detect CH_3_SeH in a coupled activity assay. The metabolite methylselenol and its oxidized form from MSC metabolism is utilized as a substrate for TrxR1 and this can be monitored spectroscopically at 340 nm. Our results show the feasibility of monitoring the β-elimination of KYAT1 by our assay and the results were compared to the previously described β-elimination assays measuring pyruvate. By using known inhibitors of KYAT1 and TrxR1, we further validated the respective reaction. Our data provide a simple but accurate method to determine the β-elimination activity of KYAT1, which is of importance for mechanistic studies of a highly interesting selenium compound.

## 1. Introduction

**Figure 1:**
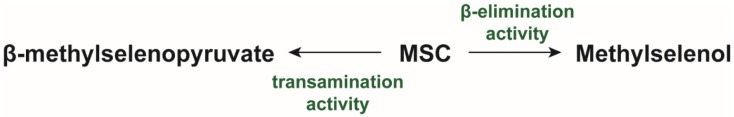
Schematic representation of β-elimination and transamination activity of the kynurenine aminotransferase 1 (KYAT1) enzyme.

Se-methylselenocysteine (MSC) is an organic methylated selenium compound that has drawn the attention of pharmacologists because of its high bioavailability, favorable pharmacokinetics and pronounced tumor-specific cytotoxic activity by altering numerous cell signaling pathways [1]. MSC itself is not an active drug, but the metabolites formed by enzymatic transformation have been reported to possess cancer prevention and anti-neoplastic properties [2]. Kynurenine aminotransferase 1 (KYAT1) plays a major role in MSC metabolism. KYAT1 is a multifunctional PLP-dependent enzyme involved in cleaving a carbon–sulfur bond, which transforms the amino acids into alpha-keto acids. This enzyme has dual activity—a transamination activity, and a beta-elimination activity. KYAT1 is mostly found in the cytosol. Se-conjugate compounds have been shown to favor beta-elimination activity over corresponding S-conjugate compounds. This may be due to a weaker C–Se bond than the C–S bond [3,4]. KYAT1 utilizes Se-methylselenocysteine (MSC) as a substrate to produce methylselenol (MS), pyruvate, and ammonium via β-elimination [5,6] and β-methylselenopyruvate (MSP) via transamination [2,7], as shown in Figure 1. Both metabolites of MSC by KYAT1, i.e., MS and MSP, possess anti-tumor and anti-proliferative properties [2,8,9,10]. Of these, methylselenol (MS) is considered an important selenium metabolite because of its selective cytotoxic effects towards tumor cells [2,11,12]. Because of this, it is necessary to measure the activity of KYAT1 when MSC is used as a substrate and to determine the relation between transamination and β-elimination. Considering the great potential of MSC as a chemotherapeutic, an accurate and simple assay method to measure the β-elimination activities in various tumor samples is of great value. Thus, the β-elimination activity of KYAT1 could be a valuable biomarker for determining which tumor would respond to MSC treatment. Methylselenol efficiently redox cycles with mammalian thioredoxin reductases (TrxR1) [13]. Thioredoxin reductase (TrxR, TR) is an important enzyme in maintaining the redox homeostasis by regulating the redox state of thioredoxin, a wide range of protein, low molecular weight thiols, and redox-active compounds. It is one of the major antioxidant systems in the cells [14]. TrxR reduces disulfides in the cell by Trx and NADPH. 

There are many simple spectrophotometric protocols available to determine the transamination activity of KYAT1 with various substrates, but there are only limited published possibilities to measure the β-elimination activity, because of its limited substrate availability and complex assay procedure. Detecting the β-elimination product from MSC is difficult because methylselenol is highly volatile and highly nucleophilic [8]. There exist few assays available to measure pyruvate, which is one of the products of the β-elimination activity of KYAT1 from MSC and it is determined by the 2, 4-dinitrophenylhydrazine (DNPH) method [5,6]. However, this is an indirect measurement of methylselenol, which measures the amount of pyruvate, i.e., it is assumed but not proven that the amount of pyruvate generated is equal to the methylselenol (1:1 ratio) generated [8]. Thus, a major problem in this assay is the uncertainty as to what is really measured. Furthermore, the DNPH method is unable to distinguish between pyruvate and other 2-oxo-acids [9]. This clearly demonstrates the need for improved assay methods. In this study, we developed a modified β-elimination assay [8] by coupling KYAT1 β-elimination with TrxR mediated reduction. 

The coupled activity assay/TrxR1 was verified with KYAT1 transamination assay and a preexisting β-elimination assay/pyruvate. Furthermore, we examined a few compounds that could be potential candidates as inducers or inhibitors of KYAT1 transamination and/or β-elimination activity. We have further validated our coupled activity assay data using these inhibitors and inducers.

## 2. Materials and Methods 

### 2.1. Chemicals and Reagents 

Potassium dihydrogen phosphate, di-potassium hydrogen phosphate, EDTA, sodium hydroxide, aminooxyacetic acid (AOAA), l-tryptophan (l-Try), indole-3-pyruvic acid (IPA), phenylpyruvic acid (PPA), α-Keto-γ-methylthiobutyric acid sodium salt (KMB), se-methylselenocysteine hydrochloride (MSC), dimethyl-2-oxoglutarate (α-KG), l-phenyl alanine (l-Phe), 2-amino-2-methyl-1,3-propanediol, pyridoxal 5′-phosphate hydrate (PLP), 2,4-dinitrophenylhydrazine (DNPH), Phenylmethanesulfonyl fluoride (PMSF), RIPA buffer, protease inhibitor cocktail mix, and *N*-*N*-dimethyl formamide, pLKO.1 vector were purchased from Sigma-Aldrich (Darmstadt, Germany). BFF-122 was purchased from Axon Medchem (Groningen, Netherlands), NADPH was purchased from Acros Organics (New Jersey, NJ, USA). Plasmid pEGFP-N1 (Clontech, Takara Bio USA, Inc, Mountain View, CA, USA) was a generous gift from Dr. Gildert Lauter from the Department for Biosciences and Nutrition, Karolinska Institutet, Stockholm, Sweden. HEPG2 cells and EMEM media were purchased from ATCC (Wesel, Germany).

### 2.2. Cell Culture 

The HEPG2 cell line was purchased from ATCC (Wesel, Germany) and maintained in EMEM (Eagle’s Minimum Essential Medium, ATCC) medium supplemented with 10% heat-inactivated Fetal Bovine Serum (FBS) (Gibco, Paisley, UK) under 5% CO_2_ at 37 °C without the addition of antibiotics. 

### 2.3. KYAT1 Over-Expression

The full-length KYAT1 coding sequence was amplified from cDNA and subsequently cloned into the pEGFP-N1 (Clontech, TakaraTakara Bio USA, Inc) mammalian expression vector. The human PGK promoter and Puromycin resistance gene sequences were amplified from pLKO.1 (Sigma Aldrich, Darmstadt, Germany ) and inserted into pEGFP-N1 backbone containing the KYAT1 expression vector. This KYAT1 over-expression plasmid was created for another ongoing study. An empty vector was created by inserting the PCR-amplified puromycin resistance gene expression cassette into pEGFP-N1 directly (manuscript in preparation).

HEPG2 cells were transfected with either the KYAT1 over-expression plasmid or the empty vector using Lipofectamine 3000 (Invitrogen, Camarillo, CA, USA) according to the manufacturer’s protocols. Briefly, cells were seeded in 60-mm dishes 24 h before transfection. The cells were 75–90% confluent at the time of transfection. Then, 2 μg plasmid was mixed with 6 μL Lipofectamine 3000 and 5 μL P3000 reagent in a total volume of 500 μL OptiMEM medium (Gibco, ) and incubated for 10–15 min at room temperature before the transfection mixture was added dropwise to the cells. The cells were harvested after 48 h of transfection and subjected to protein isolation as described in Section 2.4. 

### 2.4. Determination of Protein Concentration

Samples were lysed in ice for 30 min in RIPA buffer (Sigma, Darmstadt, Germany) in the presence of 1 mM PMSF and 1% Protease inhibitor cocktail mix (Sigma). Further, lysed cells were sonicated at 4 °C for 30 s with 1- to 2-s pulses. The proteins were harvested by centrifuging at 13,000 rpm for 10 min at 4 °C and the supernatant was collected, and protein concentration was determined by the Pierce™ Bicinchoninic acid (BCA) Protein Assay Kit (Thermo Fischer Scientific, Rockford, IL, USA) according to the manufacturer’s protocols.

### 2.5. Enzymes

KYAT1: Recombinant protein of KYAT1 (228 ng/µL), transcript variant 1 was purchased from OriGene Technologies (Rockville, MD, USA, Cat no: TP316317, Lot no: 111512).

TrxR1: Mammalian TrxR1 was purchased from IMCO (Stockholm, Sweden) and Sigma-Aldrich (Cas no:9074-14-0, Product number T9698, Darmstadt, Germany).

### 2.6. Buffer Preparation

100 mM potassium phosphate buffer contained 80.2 mL of 100 mM K_2_HPO_4_, 19.8 mL of 100 mM KH_2_PO_4_ and 2 mM of EDTA, and the pH was adjusted to 7.4 with 1 M NaOH.

The ammediol-HCL buffer contained 50 mL of 200 mM 2-amino-2-methyl-1,3-propanediol (21.03 g/L) and the pH was adjusted to 9.0 with 200 mM HCl (approximately 16.7 mL) and distilled water was added to a final volume of 100 mL.

Preparation of 10 mM DNPH; 39.6 mg of DNPH was dissolved in 10 mL of 2 M HCl.

### 2.7. Enzyme Assay

#### 2.7.1. Transamination Assay

KYAT1 transamination activity was measured with pure protein or whole-cell lysate according to the published procedure [15,16]. Briefly, 50 µL of the reaction mixture contained 20 mM of l-Phe, 5 mM of KMB, 100 mM of ammediol-HCl buffer (pH 9.0) and 50 ng to 400 ng of pure KYAT1 protein or 20 µg of protein from whole-cell lysate. The assay mixture was incubated at 37°C and the reaction was terminated by adding 150 µL of 1 M NaOH at different time points. The production of phenylpyruvate-enol was assessed by measuring the absorbance at 320 nm in a spectrophotometer (PowerWave HT, BioTek, Winooski, VT, USA). The assay mixture without enzyme was used as blank. Under this condition, the molar extinction coefficient is 16,000 M^−1^ cm^−1^ for phenylpyruvate-enol [15]. UV flat-bottom plates (Corning, Kennebunk, ME, USA) were used for this assay. To assess the effect of transamination inhibitors on the enzyme activity, the respective compounds were added at the desired concentration as indicated with 50 ng of KYAT1 enzyme. The reaction was pre-incubated for 5 min at 37 °C before being added the enzyme and KMB.

#### 2.7.2. Beta-Elimination Assay/Pyruvate

KYAT1 β-elimination activity was measured with pure protein or whole-cell extract according to the published procedure [17]. The assay protocol was adjusted to measure pure protein activity in 96-well plate readers for users’ convenience. Briefly, 50 µL of the reaction mixture contained 100 mM potassium phosphate buffer pH 7.4, 5 mM of MSC, 100 µM of αKG, 100 µM of KMB, 10 µM of PLP and 25 ng to 400 ng of pure KYAT1 protein or 20 µg of protein from whole-cell lysate. The reaction mixture was incubated at 37 °C and the reaction was terminated by adding 20 µL of 10 mM 2,4-dinitrophenylhydrazine in 2 M HCl at different time points. Then, 70 µL of this assay mixture was incubated at 37 °C for 10 min, 130 µL of 1 M NaOH was added and increases in absorbance were measured at 515 nm [18] in a spectrophotometer (PowerWave HT, BioTek). The assay mixture without enzyme was used as blank. Under this condition, the molar extinction coefficient is 16,000 M^−1^ cm^−1^ for pyruvate 2, 4-dinitrophenylhydrazone. The reaction was pre-incubated for 5 min at 37 °C before adding the enzyme. To assess the effect of β-elimination inhibitors on the enzyme activity, the respective compounds were added at the desired concentration as indicated with 200 ng of KYAT1 enzyme.

#### 2.7.3. Coupled Activity Assay/TrxR1

Our coupled activity assay combines 2 different enzyme activities to detect methylselenol production, i.e., thioredoxin reductase 1 and KYAT1. Briefly, 100 µL of reaction mixture contained 100 mM potassium phosphate buffer pH 7.4, 1–10 mM of MSC, 100 µM of αKG, 100 µM of KMB, 10 µM of PLP, 0.4 µg mammalian TrxR1, 400 µM of NADPH and 50 ng to 400 ng of pure KYAT1 protein or 20 µg of protein from whole cell lysate. The reaction mixture was pre-incubated for 5 min at 37 °C before adding TrxR1 and NADPH. Continuous measurement of NADPH consumption was recorded at 340 nm for every 30 s using a spectrophotometer (PowerWave HT, BioTek). The assay mixture without TrxR1 was used as blank. Under this condition, the molar extinction coefficient was 6220 M^−1^ cm^−1^ [19,20] for NADPH. T_max_ represents the time taken to consume all the available NADPH in the assay mixture. To assess the inhibitory effects of β-elimination inhibitors on the enzyme activity, the respective compounds were added at the desired concentration as indicated with 100 ng of KYAT1 enzyme.

### 2.8. Statistical Analysis

The results are expressed in Box and Whisker plots showing median, 25- and 75- percentiles. The analysis was performed by either student *t*-test or one-way ANOVA with 95% confidential interval followed by Dunnett multiple comparison tests (* *p* < 0.05, ** *p* < 0.01, *** *p* < 0.01 and **** *p* < 0.0001) compared to control. The data were analyzed with GraphPad Prism software, version 6 (GraphPad Software Inc, San Diego, CA, USA). 

## 3. Results

### 3.1. TrxR1 and KYAT1 Coupled Enzyme Reaction

**Figure 2:**
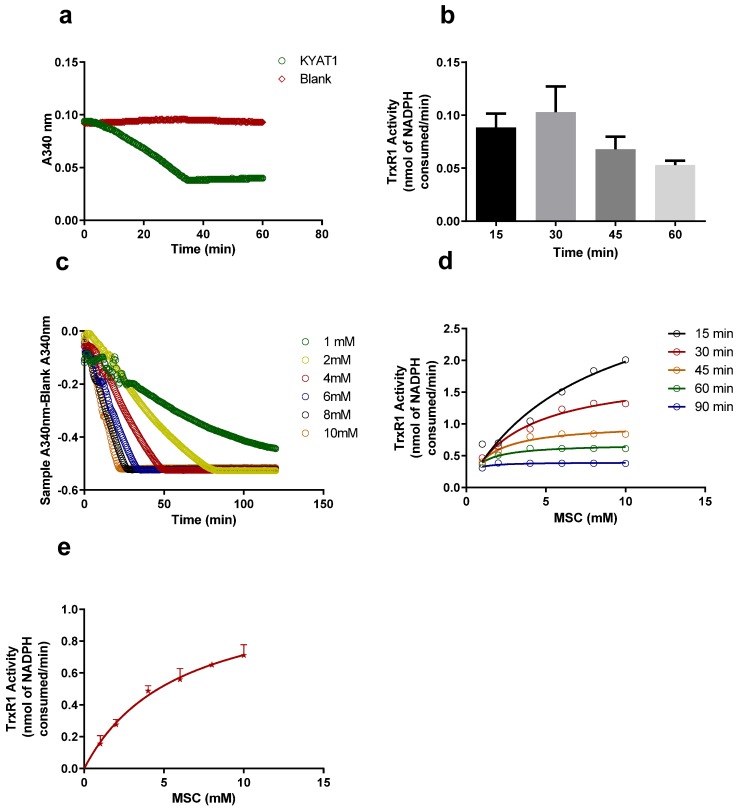
Kynurenine aminotransferase 1 (KYAT1) and thioredoxin reductase 1 (TrxR1) coupled activity assay to detect methylselenol generation. (**a**) TrxR1 activity in the coupled activity assay mixture. The assay mixture contains 5 mM Se-methylselenocysteine (MSC) as a primary substrate and 100 ng of KYAT1 as a primary enzyme to metabolize MSC into methylselenol. Methylselenol was utilized as a substrate by TrxR1, which is monitored by a decrease in absorbance at 340 nm with time in a spectrophotometer (oxidation of NADPH to NADP^+^). (**b**) The TrxR1 activity is represented in nmol of NADPH consumed/min. i.e., for 15, 30, 45 and 60 min. Data are represented as mean ± SD (*n* = 5). (**c**) TrxR1 activity with increased substrate concentration (MSC) with 100 ng of KYAT1. i.e., 1, 2, 4, 6, 8 and 10 mM of MSC, the T_max_ velocity is >120, 83.6, 53.6, 35.0, 27.0 and 24.6 min respectively. (**d**) The enzyme activity with varying concentrations of the substrate at different time points. With 100 ng of KYAT1 enzyme, the maximum activity can be observed at 15 min in all substrate concentrations. (**e**) TrxR1 activity at 30 min with increasing substrate concentration (MSC) in the presence of 200 ng of KYAT1 and 0.41µg of TrxR1. The apparent K_m_ and V_max_ for the TrxR1 coupled reaction was calculated to 5.84 ± 0.95 mM and 1.12 ± 0.08 nmol/min. Data are represented as mean ± SD (*n* = 4).

The β-elimination activity of KYAT1 with MSC as a substrate was shown by Ioanna Andreadou et al. [8] using HPLC, which measured the amount of pyruvate formed. Later, J.L. Cooper et al. [15] used the DNPH method to quantify pyruvate. In this study, we modified the J.L. Cooper et al. [15] protocol and added TrxR1 into the assay system. We could observe the consumption of NADPH by a decrease in absorbance at 340 nm (Figure 2a). This indicated that TrxR1 utilizes the product of the β-elimination activity of KYAT1 (methylselenol) as its substrate, resulting in redox cycles and NADPH consumption [21].

Figure 2b shows TrxR1 activity over time in this coupled activity assay. The following reaction is nonstoichiometric if there is access to oxygen and NADPH, i.e., most of the NADPH was consumed within 30 min. The consumption of NADPH increased with increased substrate concentration. The maximum time taken to consume all the NADPH was >120, 83.6, 53.6, 35.0, 27.0 and 24.6 min for 1,2,4,6,8 and 10 mM of MSC, respectively (Figure 2c), and Figure 2d shows the quick clearance of NADPH, i.e., there is only a little NADPH available after 45 min, so it is necessary to calculate the NADPH consumption at the end of 15 and/or 30 min with 1–6 mM MSC concentration.

### 3.2. Determination of Reaction Rate with Two Different Substrates

The coupled β-elimination assay/TrxR1 is kinetically complicated since it involves the activities of two enzymes with three different substrates i.e., MSC, NADPH, and monomethylselenol. In order to characterize the kinetics, the apparent K_m_ and V_max_ were calculated under saturated conditions. The apparent K_m_ and V_max_ were determined to 5.84 ± 0.95 mM and 1.12 ± 0.08 nmol/min respectively (Figure 2e). Under these conditions, a K_cat_ of 160 min^−1^ was calculated. We used the K_cat_/K_m_ value to calculate the turnover number or catalytic efficiency of the enzyme. Under these conditions, the turnover number was 27.4 mM^−1^ min^−1^. 

### 3.3. Comparing KYAT1 Enzyme Activity in Transamination, β-Elimination/Pyruvate, and Coupled Activity Assay/TrxR1

**Figure 3:**
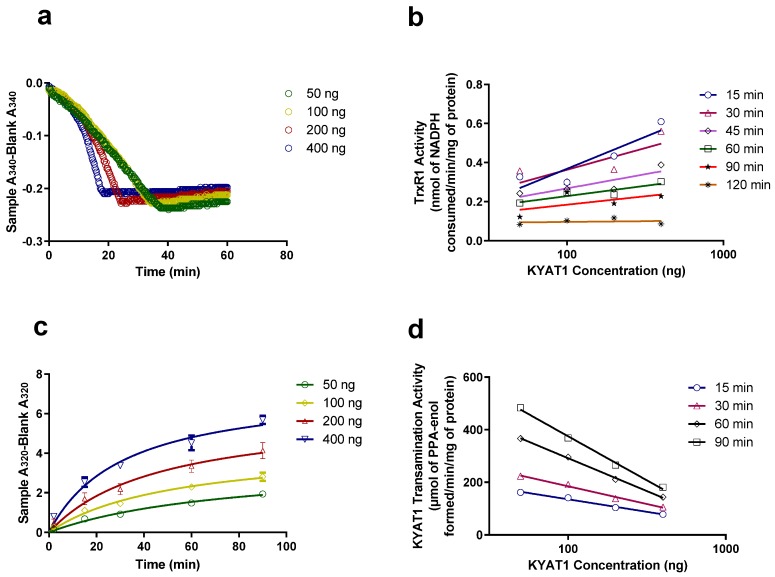
Comparison between beta-elimination and transamination activity. (**a**) Increased KYAT1 concentration increases NADPH consumption by TrxR1. The T_max_ velocity for 50, 100, 200 and 400 ng of KYAT1 enzymes resulted in 49.71, 49.68, 24.0 and 14.65 min, respectively, with 5 mM of MSC as a substrate. (**b**) The activity of the KYAT1 enzyme at varying concentrations with time in the coupled activity assay. (**c**) The transamination activity of KYAT1 at varying enzyme concentration, ½T_max_ velocity for 50, 100, 200 and 400 ng of KYAT1 enzymes resulted in 78.15, 53.0, 46.4 and 31.7 min, respectively. Data are represented as mean ± SD (*n* = 3). (**d**) The activity of the KYAT1 enzyme at varying concentrations with time in the transamination assay.

To validate our coupled activity assay, we compared the enzyme activity with varying enzyme concentrations in transamination and coupled β-elimination assay/TrxR1. Figure 3a,c show the activity of the enzyme with increasing enzyme concentration. Figure 3a is a proof of our concept that TrxR1 uses methylselenol as a substrate and consume NADPH, as the enzyme concentration increases, the NADPH consumption also increases which reflects in the T_max_ velocity. Comparable results were obtained from the transamination assay (Figure 3c). As the coupled assay relies on NADPH availability, it is important to calculate the TrxR1 activity and/or MS generation at 15 or 30 min (Figure 3b), but in case of transamination activity we determine the activity at 30 or 60 min (Figure 3d), i.e., this assay measures the formation of PPA-enol and coupled β-elimination assay/TrxR1 measures the consumption of NADPH. From Figure 3a,d, it is evident that the minimum amount of enzyme required for coupled activity assay/TrxR1 and transamination assay is 100 and 50 ng, respectively.

### 3.4. Comparing β-Elimination Activity Assay/Pyruvate with a Coupled Activity Assay/TrxR1

**Figure 4:**
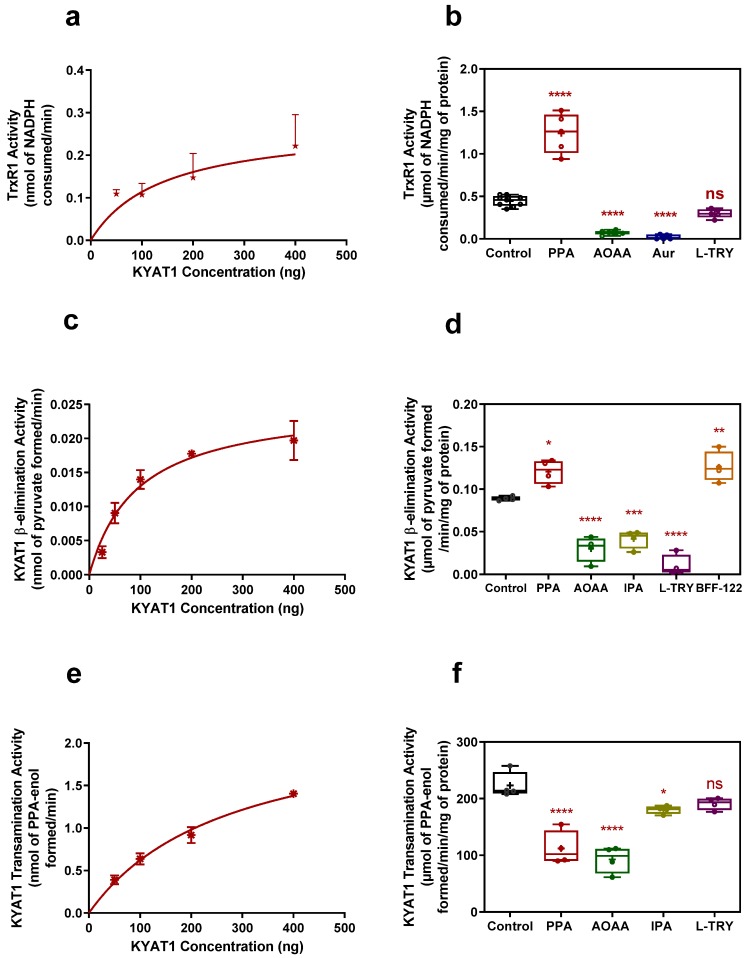
Transamination and β-elimination activity of KYAT1 with inducers and inhibitors (**a**) Coupled activity assay at 15 min, TrxR1 activity with varying KYAT1 enzyme concentration. The optimum KYAT1 concentration for maximum NADPH consumption at 15 min is 140 ng (*n* = 7). (**b**) KYAT1 activity (100ng) in coupled activity assay with phenylpyruvic acid (PPA) (400 µM), aminooxy acetic acid (AOAA) (1 mM), l-tryptophan (l-Try) (2 mM) and auranofin (Aur) (100 nM) (*n* = 5–9). (**c**) The pyruvate production with varying KYAT1 enzyme concentrations by modified β-elimination assay at 60 min. The maximum activity was shown at 95 ng of an enzyme concentration (1/2T_max_ is 0.0125 nmol of pyruvate produced by 95ng of enzyme) (*n* = 4). (**d**) KYAT1 activity (200ng) in β-elimination assay with phenylpyruvic acid (PPA) (400µM), aminooxy acetic acid (AOAA) (1 mM), indole-3-pyruvic acid (IPA) (100 µM), l-tryptophan (l-Try) (2 mM) and BFF-122 (50 µM) (*n* = 4). (**e**) Transamination activity of KYAT1 enzyme at different concentrations. The optimum KYAT1 concentration for maximum phenylpyruvate-enol formation at 30 min was 274 ng (*n* = 4–5). (**f**) Transamination activity of KYAT1 (50 ng) with phenylpyruvic acid (PPA) (400 µM), aminooxy acetic acid (AOAA) (1 mM), indole-3-pyruvic acid (IPA) (100 µM) and l-tryptophan (l-Try) (2 mM) (*n* = 4). Bar graphs in (**b**) (**d**) and (**f**) represent mean ± SD, statistical analysis performed with one-way ANOVA with 95% confidential interval followed by Dunnett multiple comparison test (ns= not significant, * *p* < 0.05, ** *p* < 0.01, *** *p* < 0.001 and **** *p* < 0.0001 compared with control).

**Figure 5:**
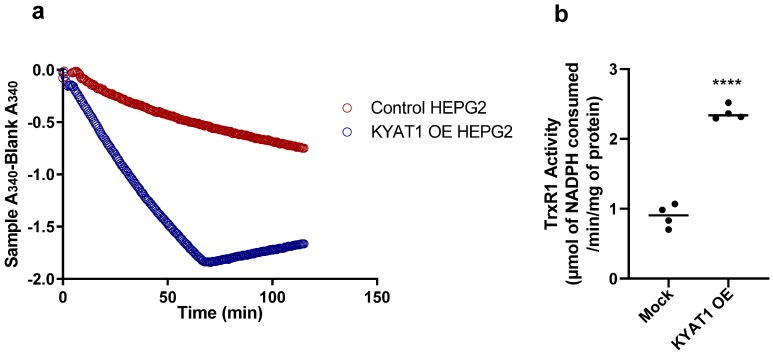
TrxR1 activity with KYAT1 over-expressed and mock transfected HEPG2 cells. (**a**) TrxR1 activity in cell lysates from control and KYAT1 over-expressed HEPG2 cells (20 µg of control and KYAT1 over-expressed cell lysate). The 1/2T_max_ velocity for control and KYAT1 overexpressed cell lysate was >120 and 48.04 min. (**b**) The TrxR1 activity at the end of 30 min for control and KYAT1 over-expressed cell lysate is 0.20 and 0.52 nmol/min or 0.90 ± 0.16 and 2.31 ± 0.10 µmol/min/mg of protein. Graph represent mean ± SD, statistical analysis performed with student t-test with 95% confidential interval (*** *p* < 0.001 compared with control).

The modified KYAT 1 β-elimination assay by J.L. Cooper et al. [15] was compared with our novel coupled activity assay for data reliability. From Figure 4a,c,e, we observed that the activity of the enzyme was linear up to 140 ng for the coupled activity assay/TrxR1, 95 ng for β-elimination assay/pyruvate and 275 ng for transamination assay. We assume that the maximum product can be calculated at this concentration of KYAT1—i.e., 0.13 ± 0.005 nmol of NADPH consumed/min, 0.011 ± 0.001 nmol of pyruvate formed/min and 1.08 ± 0.02 nmol of PPA-enol formed/min for coupled activity assay/TrxR1, β-elimination assay/pyruvate and transamination assay, respectively. To determine the feasibility of our coupled activity assay in crude-cell lysate, we transfected a KYAT1 over-expressing plasmid into HEPG2 cells and harvested these cells after 48 h. We used 20 µg of protein from the whole-cell lysate from both control and KYAT1 over-expressed cells. We observed a clear change in NADPH consumption between these two samples (Figure 5a,b). TrxR1 activity for the control and KYAT1 over-expressed cells was calculated to be 0.90 and 2.31 µmol, respectively, of NADPH consumed/min/mg of protein at the end of 30 min. This observation indicates that this coupled assay was a reliable method for both whole cell lysate and pure KYAT protein. The KYAT1 overexpression was verified by western blot (data not shown)

### 3.5. Coupled Activity/TrxR1 Assay with Different Inhibitors and Inducers

Since the coupled activity assay involves two enzyme activities, we aimed to elucidate the involvement of both enzyme activities by using inhibitors for the individual enzyme. Auranofin (Aur) at 100nM is known to inhibit TrxR1 activity completely [22]. Aminooxyacetic acid (AOAA) at 1 mM has been reported to inhibit KYAT1 activity [23,24]. Phenylpyruvic acid, a 2-oxo acid, was shown to increase the activity of the KYAT1 enzyme [9,15]. Additions of 100 nM auranofin completely abrogated the enzymatic reaction while AOAA showed a 50% decline in enzyme activity. However, the addition of 400 µM of phenyl pyruvic acid increased the reaction rate threefold. Inhibitory effects of Aur and AOAA was reproducible with the continuous coupled activity assay. Inhibitors and inducers showing 200–300% activity with PPA, 40–50% activity with AOAA, and 0–10% activity with Aur (Figure 4b) compared with control (100 ng of pure protein). These inhibitors responded similarly to the β-elimination activity assay except BFF-122 (Figure 4d). Meanwhile, KYAT1 inhibitors such as PPA, AOAA, IPA, and l-TRY showed 40–50%, 50–75%, 10–20% and 10–15% reduced enzyme activity in transamination activity assay respectively(Figure 4f). The interesting candidate from Figure 4b,d,f is PPA, which increases the β-elimination activity to threefold and inhibits the transamination activity twofold.

## 4. Discussion

Se-methylselenocysteine (MSC) is one of the most studied selenium compounds because of its high bioavailability, excellent pharmacokinetic properties and pronounced tumor-specific cytotoxicity due to its metabolites. MSC as a prodrug requires enzymatic activity to transform into an active drug. KYAT1 plays a crucial role in metabolizing MSC into an active drug. KYAT1 is highly expressed in a metabolically active organs such as the liver, kidney and brain and trace amounts of expression are also seen in the spleen, heart, large intestine, thyroid, lung and small intestine [23]. KYAT1 is mostly found in the cytosol and possesses strong aminotransferase activity towards a wide variety of amino acid substrates such as glutamine, phenylalanine, leucine, kynurenine, tryptophan, methionine, tyrosine, cysteine, asparagine and histidine [15]. Several publications have shown the anti-tumor and chemo-preventive effects of MSC metabolites produced by KYAT1, metabolites such as methylselenol and β-methylselenopyruvate (MSP) [2,25,26,27]. Although there are several assay methods available to determine the transamination product of KYAT1, there exist only a few available technically complicated indirect assays with questionable accuracy to determine the β-elimination product, i.e., methylselenol.

**Figure 6:**
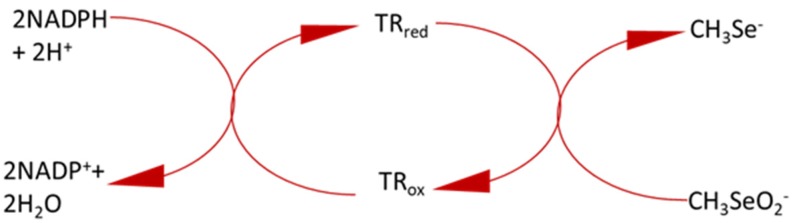
Schematic representation for the proposed mechanism of TrxR activity in reducing methylseleninate [13].

The primary goal of the present study was to establish a simple and reliable protocol to detect and measure methylselenol generation with a simple spectrophotometric method. Until now, methylselenol has been measured via laser desorption ionization-mass spectrometry (LDI-MS), GC-MS or other high throughput techniques [8,21]. The DNPH method was used to measure the β-elimination activity spectrophotometrically. This assay is an indirect measure of pyruvate and not the production of methylselenol [6]. So, in this current study, we modified the established β-elimination assay and coupled it with TrxR1. By combining these two known enzyme reactions into one assay, we created an assay detecting methylselenol generation spectrophotometrically. KYAT1 utilizes MSC as a substrate and produces methylselenol via β-elimination activity, and methylselenol reacts rapidly with oxygen to form methylseleninate (CH_3_Se^−^ + O_2_ → CH_3_SeO_2_^−^) [13]. Thioredoxin reductases utilize methylseleninate as a substrate and reduce it to CH_3_Se^-^ at the expense of 2NADPH. The following reaction was proposed by Gromer S and Gross J.H (Figure 6) [13].

NADPH + H^+^+ CH_3_SeO_2_^−^ → NADP^+^ + CH_3_SeO^−^ +H_2_O (1)

NADPH + H^+^+ CH_3_SeO^−^ → NADP^+^ + CH_3_Se^−^ + H_2_O (2)

The net reaction will be

2NADPH + 2H^+^ + CH_3_SeO_2_^−^ → 2NADP^+^ + CH_3_Se^−^ + 2H_2_O (3)

Similar TrxR1 activity with selenocystine (SeC) has previously been shown [19,28,29]. We assume that a similar kind of reaction might happen with the MSC metabolite, i.e., MS. 

During the reaction, NADPH is oxidized to NADP^+^, which is detected by a decrease in absorbance at 340 nm with time in a spectrophotometer. From the above equations, we can assume that 2 moles of NADPH result in 1 mole of CH_3_SeO_2_^−^. An increase in substrate concentration resulted in rapid NADPH consumption, i.e., higher MSC concentration results in increased MS generation. Our data clearly show that the reaction rate is proportional to the MSC concentration. The herein described coupled assay is kinetically complicated since it reflects the activities of two enzymes and three substrates. Comparisons of the apparent K_m_ and V_max_ with previously published data concerning selenite reveal that the apparent K_m_ is considerably higher (K_m_ = 5.84 mM for MSC) compared to selenite (K_m_ = 20 µM). Compared to selenocystine, the K_cat_ is much lower (160 min^−1^ for MSC and 3200 min^−1^ for SeC) [29,30]. These kinetic differences are expected due to the high reactivity of selenite and seleneocystine. MSC alone without the KYAT1 enzyme in the assay mixture does not change in absorbance compared to the blank (Figure S1a). This shows that the rate of our coupled activity assay with MSC as a substrate depends on KYAT1 metabolism. There is no literature available to show that MSP (transamination product) is a substrate for TrxR1.

The detection limit of KYAT1 in the assay was 100 ng, since there is not much difference seen between 50 and 100 ng of KYAT1. NADPH consumption was in the detectable range from 100 to 400 ng of KYAT1. NADPH consumption in the coupled assay reaction depends on the concentration of KYAT1 and substrate (MSC) respectively. Apart from the simplicity of the assay, another advantage of this assay is that it requires a minimum amount of enzyme compared to the DNPH method. But with transamination activity, KYAT1 is more competent, even at 50 ng, because it was reported in several publications that KYAT1 favors transamination over β-elimination with a 4:1 ratio [6,15] when sulfur-substrates are used, i.e., KYAT1 is more efficient in transamination of phenylalanine to phenylpyruvate-enol. For the modified β-elimination assay, we have changed the absorbance from 420 to 515, because at this wavelength we observe high linearity with pyruvic acid standard (data not shown) and reduced background noises with colored compounds [18]. So as to make sure that the change in absorbance is because of MS generation rather than protein oxidation itself, we changed the substrate from MSC to l-Phe in coupled activity assay (l-Phe is known to be an excellent substrate of KYAT1 for transamination) [15] and we did not observe any changes in absorbance (Figure S1b) compared to blank. This is clear evidence that the decrease in absorbance is because of MSC metabolism into MS. 

According to eq.3, the amount of MS can be calculated from Figure 4a, i.e., 140 ng of enzyme consumes 0.13 ± 0.005 nmol of NADPH/min, which can be written as 0.065 ± 0.0027 nmol of MS generated/min. A complicating factor is that MS can redox cycle with oxygen and a TrxR1 and thus the assay is not proportional to the amount of MSC/MS. However, our data clearly show that the consumption of NADPH was proportional to the amount of enzyme with the detection limit of 100 ng.

To further characterize and validate the method, we used chemical modifiers for TrxR1 and KYAT1 activities respectively. To show the accuracy of the assay we used both KYAT1 and TrxR1 inhibitors. Auranofin (Aur) is known to be an effective inhibitor of TrxR1 [31], and 100 nM of Aur completely inhibits the TrxR1 activity [19]. In our study, we observed a complete cessation of NADPH consumption with the addition of 100 nM Aur. On the contrary, the KYAT1 inhibitor AOAA showed a 50% reduced NADPH consumption. AOAA has been shown to inhibit pyridoxal phosphate-dependent enzymes, such as KYAT1, effectively at 1 mM [23,24]. AOAA inhibited the enzyme activity in both the β-elimination and transamination assay. Inhibition was not limited to β-elimination activity, as previously reported by Elfarra et al. (1986), and Stevens et al. (1989) [24,32]. Phenyl pyruvic acid (PPA) was shown to increase the activity of KYAT1 [9,15]. We have shown in this study that PPA increased the enzyme activity towards MSC about 3–4-fold in the coupled activity assay/TrxR1 and 1.5-fold in the β-elimination assay/pyruvate (Figure 4d). Interestingly, it specifically increased the KYAT1 β-elimination activity, whereas it partially inhibited (40–50%) the transamination activity. Other inhibitors, such as BFF-122 [33], showed 1.5-fold increased activity rather than inhibiting the enzyme, in the β-elimination assay, but not in the coupled or transamination assay. α-ketoglutarate [34] did not show any inhibitory or inducing activity towards the KYAT1 enzyme reaction in either the transamination nor β-elimination assay (data not shown); α-ketoglutarate is known to increase the KYAT1 activity [17]. Indole-3-pyruvic acid [34,35] showed a 50% reduced activity in the β-elimination activity assay and 20% reduced activity in transamination activity assay; we could not observe these effects in the coupled assay because the color of this compound interferes with the assay. As l-tryptophan is known to be a competitive inhibitor for the KYAT1 enzyme [35], we observed a strong inhibitory effect in the β-elimination/pyruvate activity assay but mild inhibitory effects in the coupled and transamination activity assay, but the data were not significant. From this data, we hypothesize that the assay described herein can be plausible in the whole-cell lysate. As expected, we found a clear deviation in NADPH consumption between control and KYAT1 over-expressed HEPG2 whole-cell lysate. TrxR1 activity for control and KYAT1 over-expressed cell lysate was, respectively, 0.9 ± 0.16 and 2.31 ± 0.10 µmol of NADPH consumed/min/mg of protein. From these data, we assume that the β-elimination activity in the control and KYAT1 over-expressed cells could be, respectively, 0.45 and 1.155 µmol/min/mg of protein at 30 min. We have observed threefold increases in pyruvate production at 120 min in KYAT1 over-expressed cell lysate compared to control in the β-elimination assay (Figure S2b), and a similar increase in transamination activity was also observed in these cell extracts (Figure S2a). 

In summary, the data presented herein show a simple and accurate method to determine KYAT1 β-elimination activity via spectrophotometer for both pure KYAT1 protein and whole-cell lysate. This assay is highly relevant for research of MSC, a selenium compound with outstanding potential for cancer prevention and treatment.

## 5. Conclusions

In conclusion, our data presented here show a new way of quantifying and detecting β-elimination activity by pure KYAT1 protein and whole cell lysate by combining KYAT1 and TrxR1. TrxR1 redox cycle with methylselenol and oxidizing NADPH to NADP^+^, which was observed spectrophotometrically at 340 nm. 

## Supplementary Materials

The following are available online at https://www.mdpi.com/2076-3921/9/2/139/s1, Figure S1: Coupled activity assay (a) With and without KYAT1 (100 ng) enzyme (b) with l-Phe and MSC as a substrate with 100 ng of KYAT1 enzyme, Figure S2: KYAT1 enzyme activity in Mock and KYAT1 overexpressed HEPG2 cells (a) Transamination activity at the end of 30 min in 20 µg of cell lysate (b) β-elimination activity at the end of 120min in 20 µg of cell lysate.
