# Supplementary material for: A Novel Assay Method to Determine the β-Elimination of Se-Methylselenocysteine to Monomethylselenol by Kynurenine Aminotransferase 1
Source: Antioxidants (Basel). 2020 Feb 5;9(2):139. doi: 10.3390/antiox9020139 (PMC7070735; doi:10.3390/antiox9020139)
Supplement: Supplementary file 1 [file antioxidants-09-00139-s001.pdf]

antioxidants

MDPI

Supplementary Materials:

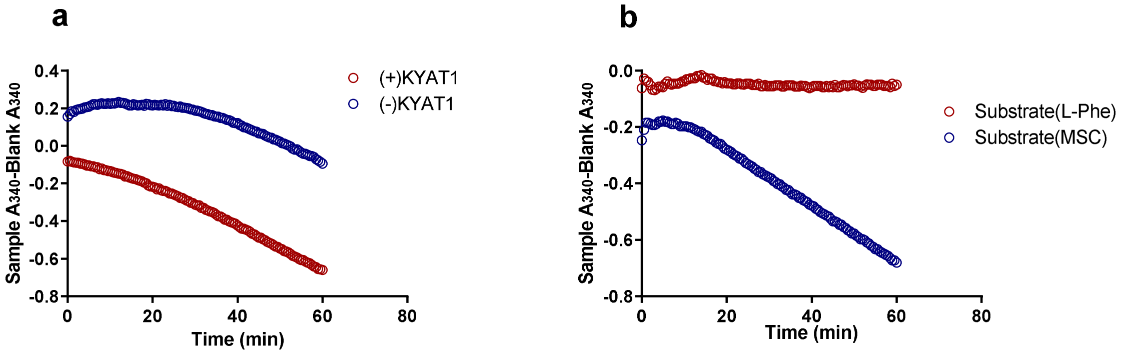

Figure S1. Coupled activity assay (**a**) With and without KYAT1(100 ng) enzyme (**b**) with L-Phe and MSC as a substrate with 100 ng of KYAT1 enzyme.

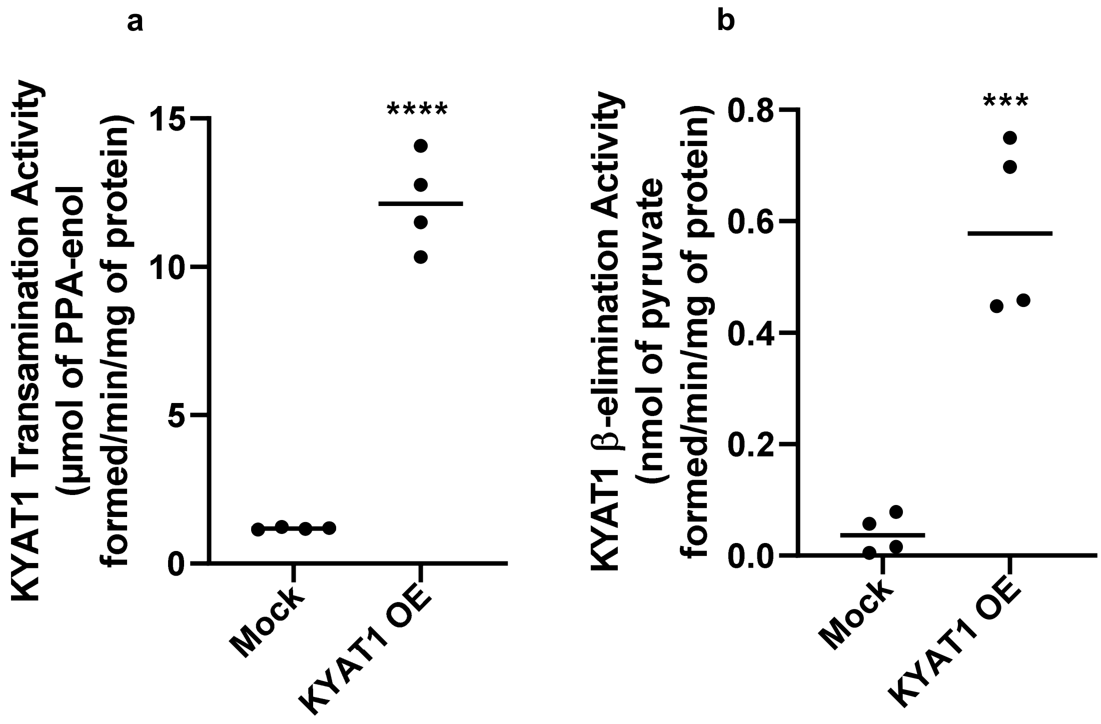

Figure S2. KYAT1 enzyme activity in Mock and KYAT1 overexpressed HEPG2 cells (**a**) Transamination activity at the end of 30 min in 20 µg of cell lysate (**b**) β-elimination activity at the end of 120 min in 20 µg of cell lysate.
